# A Circular RNA Derived from the Pumilio 1 Gene Could Regulate PTEN in Human Cumulus Cells

**DOI:** 10.3390/genes15010124

**Published:** 2024-01-19

**Authors:** Angela Caponnetto, Carmen Ferrara, Anna Fazzio, Noemi Agosta, Marianna Scribano, Maria Elena Vento, Placido Borzì, Cristina Barbagallo, Michele Stella, Marco Ragusa, Paolo Scollo, Davide Barbagallo, Michele Purrello, Cinzia Di Pietro, Rosalia Battaglia

**Affiliations:** 1 Department of Biomedical and Biotechnological Sciences, Section of Biology and Genetics “G. Sichel”, University of Catania, 95123 Catania, Italy; carmen.ferrara@phd.unict.it (C.F.); anna.fazzio@phd.unict.it (A.F.); cbarbagallo@unict.it (C.B.); michelestella7@gmail.com (M.S.); mragusa@unict.it (M.R.); dbarbaga@unict.it (D.B.); purrello@unict.it (M.P.); dipietro@unict.it (C.D.P.); rosalia.battaglia@unict.it (R.B.); 2 Department of Physics and Astronomy “Ettore Majorana”, University of Catania, 95123 Catania, Italy; 3 Department of General Surgery and Medical-Surgical Specialties, University of Catania, 95123 Catania, Italy; noemi1999gaia@gmail.com (N.A.); scribanomarianna@gmail.com (M.S.); 4 IVF Unit, Cannizzaro Hospital, 95123 Catania, Italy; mrln.vento@gmail.com (M.E.V.); pla.borzi@gmail.com (P.B.); 5 Department of Medicine and Surgery, Kore University, 94100 Enna, Italy; paolo.scollo@unikore.it; 6 Maternal and Child Department, Obstetrics and Gynecology Unit, Cannizzaro Hospital, 95123 Catania, Italy

**Keywords:** circRNAs, ovarian follicle, female gametogenesis, PTEN, PUM1

## Abstract

CircRNAs are a class of non-coding RNAs able to regulate gene expression at multiple levels. Their involvement in physiological processes, as well as their altered regulation in different human diseases, both tumoral and non-tumoral, is well documented. However, little is known about their involvement in female reproduction. This study aims to identify circRNAs potentially involved in reproductive women’s health. Candidate circRNAs expressed in ovary and sponging miRNAs, already known to be expressed in the ovary, were selected by a computational approach. Using real time PCR, we verified their expression and identified circPUM1 as the most interesting candidate circRNA for further analyses. We assessed the expression of circPUM1 and its linear counterpart in all the follicle compartments and, using a computational and experimental approach, identified circPUM1 direct and indirect targets, miRNAs and mRNAs, respectively, in cumulus cells. We found that both circPUM1 and its mRNA host gene are co-expressed in all the follicle compartments and proposed circPUM1 as a potential regulator of PTEN, finding a strong positive correlation between circPUM1 and PTEN mRNA. These results suggest a possible regulation of PTEN by circPUM1 in cumulus cells and point out the important role of circRNA inside the pathways related to follicle growth and oocyte maturation.

## 1. Introduction

In the last few decades, there has been a spread of large-scale genome sequencing studies that have revealed that only 2% of the mammalian genome produces translated RNAs (mRNAs), while the remaining part is transcribed into non-coding protein RNAs (ncRNAs) [1]. Originally, this portion of the human genome was considered a by-product of massive transcription with less biological meaning. However, today, it is commonly thought that ncRNAs have many important regulatory functions [2]. They participate in multiple biological pathways, regulating physiological and pathological processes [3,4,5,6,7]. NcRNAs may be classified according to their function into the following: housekeeping ncRNAs (rRNAs, tRNAs, small nuclear RNAs, small nucleolar RNAs, and telomerase RNAs) and regulatory ncRNAs. The last ones, depending on their lengths, are further categorized into small non-coding RNAs (ranging from 50 to 200 nt) and long non-coding RNAs (more than 200 nt). A particular class of regulatory ncRNA is the circular RNAs (circRNAs) [8]. CircRNAs are characterized by a peculiar circular shape, lacking free terminal structures, derived from non-canonical splicing events of linear pre-mRNA (back-splicing) that generate 3′ and 5′ ends covalently joined together. This peculiar round structure promotes their stability and protects them from degradation using RNA exonucleases or RNase R [9]. Usually, circRNAs are expressed at low levels and show cell type- or tissue-specific expression, indicating their potential regulatory roles [10]. Their ability to sponge microRNAs (miRNAs), acting within the competing endogenous RNA (ceRNA) network, is relatively well known. Based on this system, a specific circRNA can impair miRNA activity, influencing the expression of mRNA miRNA targets [11,12]. Moreover, circRNAs can regulate the expression of their host genes using the interaction with RNA polymerase II; they can interact with RNA-binding proteins, and some of them can be translated into proteins. Their involvement in physiological processes as well as their altered regulation in different human diseases is well documented [13,14,15,16].

Cumulus cells (CCs) are a subset of granulosa cells (GC) surrounding oocytes. The existence of an oocyte–cumulus cell regulatory loop is necessary for follicle development and the acquisition of oocyte competence.

Various papers have shown the importance of regulatory networks based on ncRNAs in human oocytes and CCs and, in particular, the involvement of circRNAs in regulating molecular functions related to human reproduction [17,18,19,20,21]. CircRNAs have been characterized by high-throughput sequencing in human CCs, and they have been proposed to be potentially involved in endometriosis-associated infertility [22] and in polycystic ovary syndrome (PCOS) [20,23,24].

In animal models, circRNA expression has been well characterized in the ovary (GCs, CCs, and oocytes), in the testes, placenta, and embryo, and also in germ line stem cells, embryonic stem cells, and induced pluripotent stem cells [25]. However, only in a few cases has the regulatory role of circRNAs been functionally demonstrated [26]. Cao Z. and colleagues demonstrated for the first time in 2019 that circRNAs are dynamically and highly expressed in a developmental stage-specific manner in CCs and oocytes and that maternally expressed circRNAs are essential for porcine oocyte meiotic maturation and early embryo development [25].

**Figure 1:**
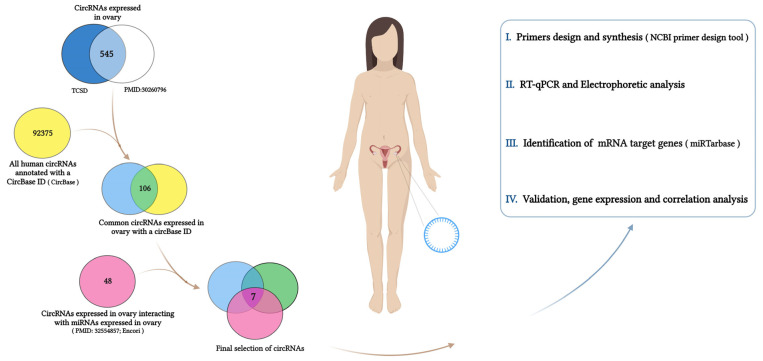
Workflow edited by BioRender “https://www.biorender.com/ (accessed on 20 November 2023)”.

In this study, using different approaches, we proved the expression of the little-known circPUM1 specific isoform in the different compartments of a mature human ovarian follicle (Figure 1). This circRNA arises from the PUM1 gene, a member of the Pumilio RNA-binding protein family. PUM family proteins are ubiquitously present across eukaryotes, including yeast, plants, and humans, and perform their regulatory role at post-transcriptional/translational levels [27]. The high evolutionary conservation of the RNA-binding domain and functions suggest that human proteins play a crucial role in the translational regulations of embryogenesis, cell development, and differentiation [27]. We verified the co-expression of circPUM1 with the mRNA of its host gene and, using a computational and experimental approach, identified circPUM1 as a potential regulator of PTEN, finding a strong positive correlation between circPUM1 and PTEN mRNA. These results suggest the possible regulation of PTEN by circPUM1 in CCs and point out the important role of circRNA in the female reproduction system.

## 2. Materials and Methods

### 2.1. Selection of circRNAs Expressed in the Ovary

In order to pinpoint the circRNAs performing a significant role inside the ovarian follicle and to understand their biological functions, we intersected different public databases, performing a bioinformatic analysis, as described below and in Figure 1.

Using the Tissue-Specific CircRNA Database (TSCD) “http://gb.whu.edu.cn/TSCD (accessed on 10 February 2023)”, we obtained a list of circRNAs specifically expressed in the ovary. This list of circRNAs was matched to high-throughput sequencing analysis of circRNAs in normal human ovaries by Hongcai Cai et al., 2018 [28]. We considered the common circRNAs and selected only the circRNAs annotated in the circBase database “http://www.circbase.org/ (accessed on 12 February 2023)”. In order to detect circRNA–miRNA interactions significant in the ovary, in parallel, we compared the list of circRNAs expressed in the ovary (Figure 1) with the list of circRNAs sponging miRNAs expressed in the ovary [29]. This last list was obtained by ENCORI (Encyclopedia of RNA Interactomes) “https://rnasysu.com/encori/ (accessed on 12 February 2023)”, which identified the interactions, supported by CLIP-seq data, between miRNAs already known to be expressed in the ovary [29] and all human-annotated circRNAs.

### 2.2. Patient Enrollment

Participants enrolled in this study were women having undergone ovarian stimulation protocol and intracytoplasmatic sperm injection (ICSI) at the IVF Center Cannizzaro Hospital Catania (Italy). Healthy women whose primary infertility was caused by male factors were included in the study; those with pathologies that could influence oocyte quality, such as endometriosis, polycystic ovary, and ovarian insufficiency, were excluded from the study, as well as heavy smokers and overweight women.

Our research followed the tenets of the Declaration of Helsinki. The patients gave their informed consent to use their CCs and follicular fluid (FF). Women undergoing infertility treatment and already having a successful pregnancy donated the supernumerary oocytes. These oocytes were vitrified after their collection and thawed for our research application.

### 2.3. Sample Collection

CCs, FFs, and oocytes were independently collected. CC samples were collected according to the protocol reported by Caponnetto et al., 2022 [21]. Three to seven CC samples per patient were collected from the oocytes mechanically using needles and set aside individually. The total removal of the cumulus and corona cells continued using both enzymatic (hyaluronidase) and mechanical (pipetting) methods in order to identify oocytes in metaphase II (MII). Only the cumulus cells from the MII oocytes were washed in the culture medium and frozen at −20 °C.

The collection of FF samples was performed as reported by Caponnetto et al., 2021 [5]. Briefly, in order to remove residual follicular cells and any blood traces, FF samples were centrifuged for 20 min at 1000× *g* at 4 °C; the supernatant was collected and stored at −20 °C until use. Only FF samples without any massive blood contaminations and in which nuclear mature oocytes (MII) were identified were included in the study.

At the end of the injection, the oocyte supernumeraries for the fertilization were vitrified. After the decision of the couples to donate surplus oocytes for research, 3 oocytes coming from 2 different patients were thawed and used for molecular applications. Commercially available total RNAs from physiological brain, heart, and liver tissues were obtained from Ambion (Austin, TX, USA).

### 2.4. RNA Isolation and Purification

Total RNA isolation and purification from CC samples were performed by TRIzol^®^ (Thermo Fisher Scientific, Waltham, MA, USA), according to the manufacturer’s instructions. Total RNA from FF samples was isolated as follows: RNA was isolated separately from two aliquots of 200 μL of the same FF sample by using the Qiagen miRNeasy Mini Kit (Qiagen, GmbH, Hilden, Germany), according to Qiagen Supplementary Protocol, for the purification of the RNA (including small RNAs) from serum or plasma. The two aliquots of the same FF sample were mixed together in the final step of the procedure. RNA precipitation protocol was performed in order to increase the final yield of RNA. Briefly, RNA was eluted in 200 μL of RNAse-free water; combined with 20 μg of UltraPure Glycogen (Thermo Fisher Scientific, Waltham, MA, USA), 0.1 volumes of 3 M sodium acetate, and 2.5 volumes of ice-cold absolute ethanol; and incubated at –80 °C overnight. RNA was then washed three times in ice-cold 75% ethanol, resuspended in 7 μL of RNAse-free water, and stored at –80 °C for further analyses.

Total RNAs from CCs and FFs were quantified by a spectrophotometer. Samples showing a 260–280 ratio absorbance of approximately 2.0 were considered.

According to previously published protocols, RNA from human oocytes was extracted by thermolysis; the samples were incubated for 1 min at 100 °C in order to release nucleic acids [30,31].

### 2.5. Gene Expression Analysis

In order to amplify circRNAs and mRNAs, primer design was performed by using the NCBI primer blast tool “https://www.ncbi.nlm.nih.gov/tools/primer-blast/ (accessed on 30 March 2023)”. For the detection of circular isoforms, divergent primers were designed; convergent primers were used for the detection of linear mRNAs (Table 1). Primer pairs were tested in silico, and all those recognizing more than one circular isoform produced by the same host gene were discarded. Only in the case where it was not possible to obtain primer pairs for the specific circRNA isoform, two primer pairs were designed and tested. All specific circRNA primer pairs were tested in human CCs, as well as the expression of the circRNAs selected.

Total RNA was amplified in a one-step reaction by the RNA-to-Ct™ 1-Step Kit (Thermo Fisher Scientific, Waltham, MA, USA) using convergent and divergent primers already mentioned and reported in Table 1.

MiRNA gene expression analysis was performed by using single TaqMan microRNA assays. Total RNA was reverse transcribed through the TaqMan™ microRNA Reverse Transcription Kit (Thermo Fisher Scientific, Waltham, MA, USA) using miRNA-specific primers and then amplified by the TaqMan™ Universal Master Mix II (Thermo Fisher Scientific, Waltham, MA, USA) by using specific TaqMan™ assays, according to the manufacturer’s instructions. 

All quantitative RT-qPCR reactions were performed on the QuantStudio™ 7 Flex Real-Time PCR system (Thermo Fisher Scientific), according to the supplier’s protocol. GAPDH was used as the endogenous control gene for the normalization of linear and circular transcripts [32,33,34,35]. RNU6 was used as the endogenous control for miRNA gene expression data normalization.

### 2.6. Prediction of circRNA–miRNA–mRNA Interactions

After the identification of possible miRNAs interacting with the circPUM1 isoform (as reported in Section 2.1), we identified the mRNAs targeted by these miRNAs using the miRTarbase 9.0 beta database “https://miRTarBase.cuhk.edu.cn/ (accessed on 20 March 2023)” [36]. We queried the database by selecting only the validated bindings assessed by the reporter luciferase assay experiment.

### 2.7. Statistical Analysis

Pearson’s correlation coefficient (*r*) was used to evaluate the correlation between the expression of circPUM1 and its linear counterpart and circPUM1 and its direct and indirect interactors. Statistical significance was assessed by setting a *p*-value ≤ 0.05 as the cut-off.

### 2.8. Genomics of circPUM1

The sequences of human circPUM1 were retrieved from circBase “http://www.circbase.org/ (accessed on 1 April 2023)”. The chromosome, the nucleotide position, the genomic and spliced sequence lengths, and the gene symbol were determined by the Circular RNA Interactome database “https://circinteractome.nia.nih.gov/index.html (accessed on 1 April 2023)”.

## 3. Results

### 3.1. CircRNA Selection

The comparison between the list of circRNAs specifically expressed in ovaries from TSCD and the circRNAs expressed in normal ovaries reported by Hongcai Cai et al., 2018 [28] resulted in a list of 545 common circRNAs, 106 of which were annotated in the circBase database. Among them, seven circRNAs were potentially able to interact with miRNAs highly expressed in the ovary [29]. Table 2 shows the selected circRNAs, their host genes, and the miRNAs interacting with them.

### 3.2. CircRNA Expression

The amplification of the seven circRNAs revealed that (i) all circRNAs were expressed in human CC samples and showed Ct values ranging from 26 to 39 (Figure S1A), and (ii) amplified products showed only a single peak and non-specific amplification or primer dimer formation (Figure S1B). Only hsa_circ_0077150, hsa_circ_0004079 (1), hsa_circ_0004079 (2), and hsa_circ_0011233 showed Ct values ≤ 33 (Figure S1A,C). circPUM1 (hsa_circ_0011233) was selected for further analyses, since it showed a lower Ct value and was the most efficiently amplified (Figure S1).

### 3.3. Genomics of circPUM1

circPUM1 (hsa_circ_0011233) originates from the PUM1 host gene (Pumilio RNA-binding family member 1). This gene encodes a member of the PUF family, evolutionarily conserved RNA-binding proteins related to the Pumilio proteins of *Drosophila,* and the fem-3 mRNA-binding factor proteins of *Caenorhabditis elegans*. PUM1 (chr1: 30931506-31065717 GRCh38/hg38) is composed of 22 exons, and two spliced transcript variants encoding different isoforms have been described: NM_014676.3 and NM_001020658.2. PUM1 is the host gene of 62 circRNA isoforms, as annotated in the circBase database [8,10,37,38,39]. Among these, our isoform of circPUM1 is an exonic circRNA, 527 nt long, originating from the 15th, 16th, and 17th exons of the NM_0014676.3 transcript by a back-splicing process (Table 3 and Figure S2).

### 3.4. CircPUM1 and Its Linear Counterpart Are Expressed in the Follicular Microenvironment

**Figure 2:**
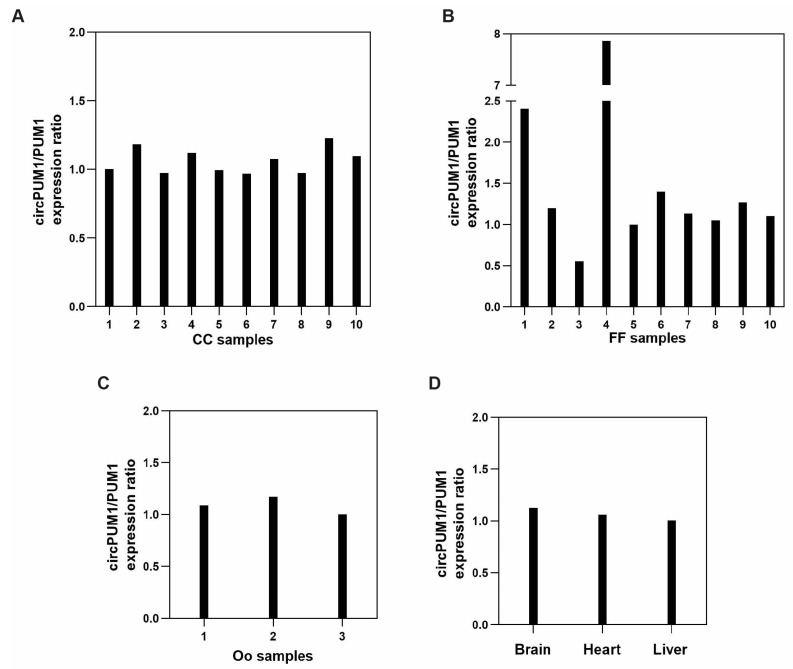
circPUM1 and PUM1 mRNA expression in ovarian follicle compartments: CCs (**A**), follicular fluids (FF) (**B**), oocytes (**C**), and other tissues (**D**) represented as the ratio of −ΔCt values.

**Figure 3:**
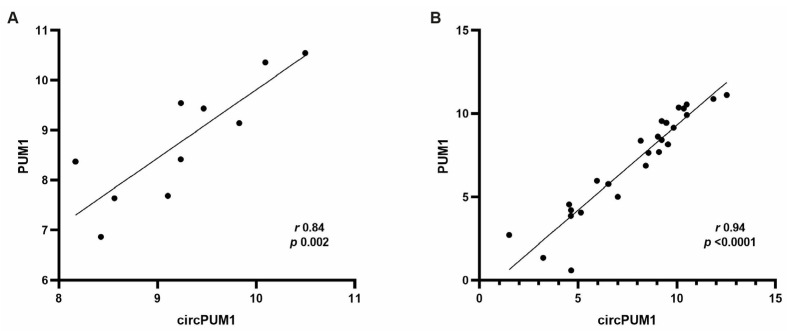
Expression changes in circPUM1 correlate with the linear transcript of PUM1 in CC samples (**A**) and all considered samples (**B**).

CircPUM1 and its linear counterpart are expressed in the follicular microenvironment, as well as in three different physiological tissues: the brain, heart, and liver. A similar expression trend, reported as the ratio circPUM1/PUM1, was observed in CC samples, oocytes, and the other tissues (Figure 2A,C,D); otherwise, FF samples showed a higher variability of circPUM1 and PUM1 expressions (Figure 2B). A significant and positive correlation between circPUM1 and PUM1 linear transcript in CCs (Pearson’s correlation coefficient: *r* = 0.84, *p* = 0.002) (Figure 3A) was observed. The correlation was stronger when all samples were considered together (*r* = 0.94, and *p* < 0.0001) (Figure 3B).

### 3.5. CircPUM1 and Its RNA Interactors in CC Samples

The computational analysis revealed that miR-16-5p and miR-214-3p were potentially sponged by circPUM1. We experimentally assessed that miR-16-5p and miR-214-3p were expressed in our CC sample cohort and identified their validated targets using the miRTarbase. As expected, the miRTarbase returned several mRNA targets for miR16-5p and miR-214-3p, among which we selected BCL2, PTEN, and TP53. In particular, BCL2 was selected, since its interaction with miR-16-5p was demonstrated by both strong and less-strong evidences and was assessed by several studies reported in literature. For the same reasons, PTEN was chosen as the interactor of miR-214-3p. Moreover, the proteins encoded by these mRNAs, as well as the TP53 protein, perform important and well-known functions in folliculogenesis [36]. 

**Figure 4:**
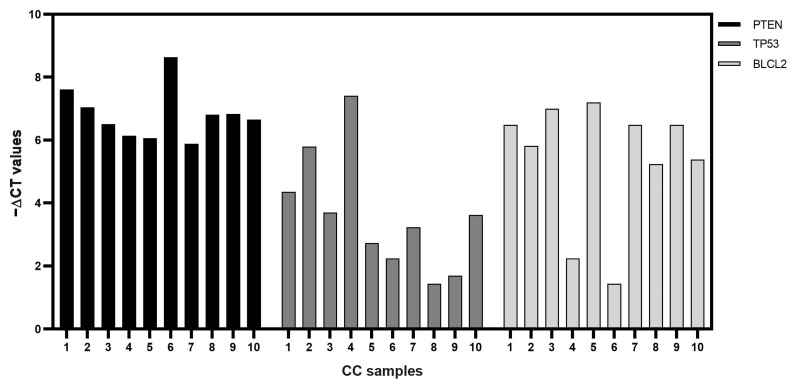
Expressions of PTEN, TP53, and BCL2 mRNAs in CC samples. Data are shown as −ΔCt values. In the x-axis, the number of CC samples is reported.

Among these, we focused our attention on BCL2, PTEN, and TP53. In particular, both miRNAs shared two of them, BCL2 and TP53, while PTEN is only targeted by miR-214-3p. All the mRNA targets selected were found to be expressed in our studied CC cohort (Figure 4).

### 3.6. Correlation between circPUM1 and mRNA Expression Levels in CCs

**Figure 5:**
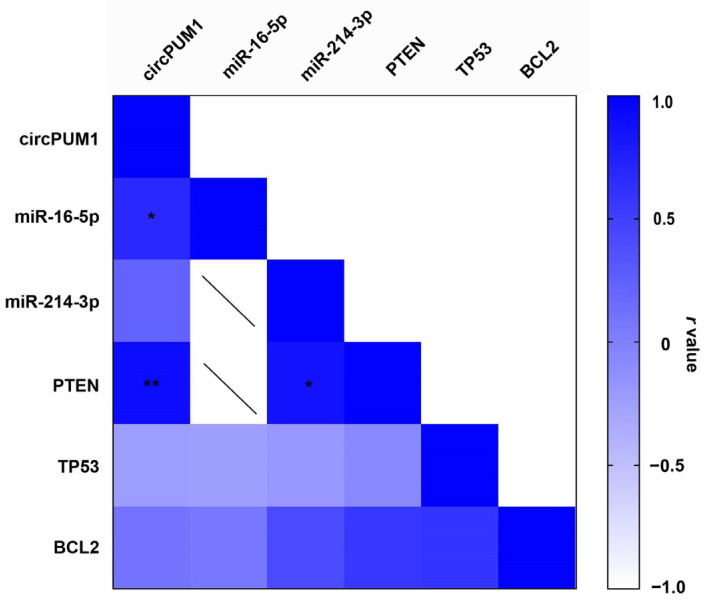
Correlation matrix among circPUM1, miRNAs 16-5p and 214-3p, and their target genes. The white–blue color scale bar indicates r values. Statistically significant correlations are reported as “*” and “**” when *p* < 0.05, and *p* < 0.01, respectively.

**Figure 6:**
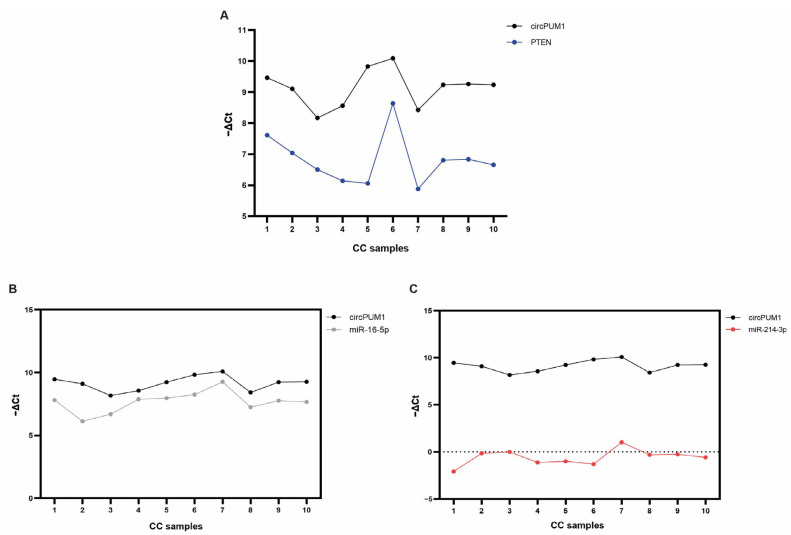
Expression trend of PTEN (**A**), miR-16-5p (**B**), and miR-214-3p (**C**) with respect to circPUM1. Data are shown as −ΔCt values. In the x-axis, the number of CC samples is reported.

In our CC samples, we found a strong positive correlation between circPUM1 and PTEN (*r* = 0.92, *p* = 0.004) (Figure 5 and Figure 6A) and also between miR-214-3p and PTEN (*r* = 0.86, *p* = 0.016). A significant positive correlation between circPUM1 and miR-16-5p was observed (*r* = 0.68, *p* = 0.04) (Figure 5 and Figure 6B). However, there was no significant correlation between circPUM1 and miR-214-3p expressions in CCs (Figure 5 and Figure 6C).

## 4. Discussion

In recent years, the discovery of novel ncRNAs has been greatly increased by high-throughput sequencing. Advances in sequencing technology and library preparation protocols, as well as computational biology have increased our knowledge about the presence of ncRNAs in prokaryotes and eukaryotes [40]. RNA sequencing provides researchers with insights into transcriptome in a cell-specific or tissue-specific manner and in different states of cellular differentiation [40,41]. However, in order to further investigate the biological and functional roles of RNA molecules, experimental validation using biochemical techniques and functional assays are needed, revealing the crucial roles of many ncRNAs in human health and disease [40,42].

Our study, using computational and experimental approaches, allowed us to identify a little-known circPUM1 isoform that could potentially play an important regulatory role inside ovarian follicles, controlling follicle maturation and meiosis resumption (Figure 1).

Several circPUM1 isoforms have already been identified in different cellular models, and their roles in the regulation of gene expression inside specific cellular processes have been demonstrated [43,44,45,46,47,48,49]. However, to date, little is known about the hsa_circ_0011233 isoform identified in this study.

The host gene, PUM1, is a member of the Pumilio RNA-binding protein family, evolutionarily conserved across species. In the human genome, we found two PUM genes closely related to each other, PUM1 and PUM2, spanning about 150 kb (on chromosome 1p35.2) and 80 kb (on chromosome 2p23–24), respectively. The indistinguishable RNA-binding specificities of PUM1 and PUM2 suggest potential overlapping functions [50]. PUM1 regulates several biological processes, such as embryonic development, functioning and development of the nervous system, rRNA processing, ribosome biogenesis, chemotactic cell movement, and also stem cell and germ cell maintenance [27]. The maintenance and self-renewal of stem cells might be an ancestral function of Pum, as demonstrated in *Drosophila* [51], *Plasmodium falciparum* [52], and zebrafish [53], as well as other model organisms. In mice, it has been demonstrated that maternal PUM1 is an essential post-transcriptional regulator during mammalian embryogenesis, regulating the stability of maternal mRNAs. PUM1 has been found to be expressed in oocytes of different organisms, such as *Xenopus* [54] and zebrafish [55], and it is involved in the control of meiosis. One of the mechanisms of action could be the post-transcriptional regulation of the mRNA of Cdk1, the kinase that dimerizes with cyclin B1, forming the metaphase-promoting factor (MPF) [56]. Moreover, knock-out experiments have revealed that PUM1 is essential in the mammalian germline, such as in the establishment of the primordial follicle pool, meiosis, and female reproductive competency, while PUM2 does not have a detectable function in these processes [57].

Here, we have demonstrated that circPUM1 is expressed in the ovarian follicle microenvironment, specifically in CCs and oocytes and also in FF samples (Figure 2). We found a strong and positive correlation between circPUM1 and its linear isoform (PUM1 mRNA), not only in the ovary but also in different tissues (Figure 3). Indeed, in FF, we found a higher variability between our circRNA and its linear counterpart, which could be expected because in biological fluids, the expression levels of RNA transcripts are affected by the secretion mechanisms and do not depend exclusively on their transcriptional levels inside the cells. However, the inclusion of FF samples in the correlation analysis, shown in the Figure 3B, did not negatively affect the result.

These data suggest that our circRNA isoform could perform similar functions as the linear counterpart and could play synergistic roles in regulating specific cellular processes involved in follicle growth and oocyte maturation. The synergic roles of circRNAs and their host genes have already been described in literature [58].

In addition, the correlation with their linear counterparts could be dynamically regulated, and the regulation of circRNAs on their corresponding host genes is a critical mechanism for their function [58]. In this regard, it is important to note that in some regulatory circuits, circRNAs and their host genes can be part of feedback loops. Changes in the expression of one component (circRNA or host gene) can influence the expression of the other, contributing to dynamic regulatory mechanisms [58]. Evaluating the expressions of PUM1 mRNA and the circRNA could be interesting under specific conditions that impair oocyte quality. 

Another important characteristic supporting the potential role of circPUM1 inside the ovarian follicle is the strong positive correlation with PTEN (phosphatase and tensin homolog) mRNA (Figure 5 and Figure 6A). PTEN is a well-known tumor suppressor gene that plays a significant role in ovarian follicle development, GC function, and overall female reproductive health. PTEN is an indispensable molecule that maintains the dormancy of the primordial follicle pool, inhibiting the PI3K signaling pathway that controls primordial follicle survival and activation; regulates cyclic follicular recruitment; causes ovulation in GCs; and stimulates meiosis resumption in the oocyte [59,60]. PTEN down-regulation is an important step in the cyclical activation of primordial follicles. The depletion of the primordial follicle pool leads to the end of female reproductive life, and it has been demonstrated that in mice lacking PTEN, the entire primordial follicle pool becomes activated, causing premature ovarian failure [61,62]. Dysregulation of PTEN in the ovaries can also have implications for fertility, ovarian disorders, and even ovarian cancer [63]. Recently, it has been demonstrated that in human GCs, PTEN expression is associated with *In Vitro Fertilization* (IVF) outcomes [64].

The relationship between circPUM1 and PTEN implies common mechanisms of regulation that could be mediated by miRNAs. However, although we found a strong and positive correlation between circPUM1 and PTEN, we did not observe any correlation between circPUM1 and miR-214-3p (Figure 5 and Figure 6C). It is important to note that the interaction between miRNAs and circRNAs within the ceRNA regulatory mechanism decreases miRNA availability, promoting target gene expression without necessarily changing miRNA levels [65]. However, the regulation of PTEN by circPUM1 could be due to a different network of regulation not mediated by miR-214-3p. Different miRNAs or other ncRNAs could be involved in this mechanism of regulation. 

Unexpectedly, we found a strong and positive correlation between miR-214-3p and PTEN (Figure 5). The positive correlation between a miRNA and its target genes has been described in several cancer models, and specifically, a positive correlation between PTEN and miR-214 has been found in breast cancer [66,67]. Recent studies indicate that miRNAs could also be implicated in the positive regulation of gene transcription by a phenomenon known as RNA activation (RNAa) [68].

In addition, we did not find any correlations between circPUM1 and the validated targets of miR-16-5p, BCL2 and TP53 (Figure 5), although we found a strong and positive correlation between circPUM1 and miR-16-5p (Figure 5 and Figure 6B). These data could suggest that circPUM1 does not play any regulatory role in the expressions of BCL2 and TP53, even if it could share regulatory mechanisms with miR-16-5p. However, miR-16-5p could exert its function inhibiting the translation of the targets without any effect on transcript degradation.

It is important to underline that our data were obtained in a physiological model and allowed us to identify a little-known cirRNA isoform, derived from the PUM1 gene and able to regulate PTEN. We propose that this study, suggesting circPUM1 as a possible regulator of PTEN, represents an important starting point for further investigations in order to understand the complex molecular mechanisms involved in reproductive women’s health. Further studies investigating the relative expressions of PUM1 and its interactors or regulators in female disorders will be needed.

## 5. Conclusions

The field of circRNA research in female reproduction is still relatively young, and ongoing studies continue to provide new insights into the specific roles and mechanisms of circRNAs in these processes. The complexity of circRNA functions and their interactions with other biomolecules make this area of research both exciting and challenging for scientists in the field of reproductive biology.

## Figures and Tables

**Table 1:** Primer sequences.

#	Gene ID	Host Gene	Forward Primer Sequence	Reverse Primer Sequence	Product Length (bp)
1	hsa_circ_0088943	NUP188	TGAAACTGGCATTCTCCGTCA	GGGCACTGAAGTAGCATGTTG	107
2	hsa_circ_0077150	PHIP	CATCACTACAGGTGAAGTATTACAG	CTCCAGGAACACTTTGAGGAAT	111
3	hsa_circ_0077147	PHIP	TCTGGGATGCTGGAACCCTT	AGTCATCAGAACCCAGCACTAA	112
4	hsa_circ_0004079 (1)	TRIP12	CACACGCCAAAAGACCACG	TAGACTGCACTTTGGGTGCC	125
	hsa_circ_0004079 (2)		GCTAGTACCAGGTCACATTTAG	TTAGACTGCACTTTGGGTGC	98
5	hsa_circ_0068888	WHSC1	AGTGTCGGGTTACCCTTGGT	CACAGAAAAGCAGACAGCTCG	271
6	hsa_circ_0011233	PUM1	GAGGCCACGTCCTGTCATTG	TGAGTCCTCCTGCTGGTCTGA	95
7	hsa_circ_0043064 (1)	MYO1D	CCTGAAGGCAAACTGAGCAT	GCACAATCACTCGAGACTTTGA	121
	hsa_circ_0043064 (2)		TACAGAGGTGACCAAGCGAC	CTCCTGGCTGTTGCACAATC	113
8	GAPDH	GAPDH	TGCACCACCAACTGCTTAGC	GGCATGGACTGTGGTCATGAG	87
9	linear PUM1	PUM1	CCTTCAGACCAGCAGAATGAGA	ATTGCAAAGACTGGGGCTGT	127
10	BCL2	BCL2	CATGTGTGTGGAGAGCGTCAA	GCCGGTTCAGGTACTCAGTCA	83
11	PTEN	PTEN	GACAATCATGTTGCAGCAATTCA	CCCATAGAAATCTAGGGCCTCT	123
12	TP53	TP53	GAGCACTGCCCAACAACAC	GTCTGGTCCTGAAGGGTGAA	85

**Table 2:** Selected circRNAs.

#	circRNA ID	Host Gene	Host Gene ID	miRNAs
1	hsa_circ_0088943	Nucleoporin 188	NUP188	hsa-miR-16-5p hsa-miR-214-3p
2	hsa_circ_0077150	Pleckstrin homology domain Interacting protein	PHIP	hsa-miR-16-5p
3	hsa_circ_0077147			hsa-miR-16-5p
4	hsa_circ_0004079 (1)	Thyroid hormone receptor interactor 12	TRIP12	hsa-miR-372-3p hsa-miR-18a-5p hsa-miR-214-3p
	hsa_circ_0004079 (2)			
5	hsa_circ_0068888	Wolf–Hirschhorn syndrome candidate 1	WHSC1	hsa-miR-16-5p hsa-miR-214-3p
6	hsa_circ_0011233	Pumilio RNA-binding family member 1	PUM1	hsa-miR-16-5p hsa-miR-214-3p
7	hsa_circ_0043064 (1)	Myosin ID	MYO1D	hsa-miR-16-5p
	hsa_circ_0043064 (2)			

**Table 3:** Information from the CircInteractome database (https://circinteractome.nia.nih.gov/index.html (accessed on 20 November 2023)).

circRNA ID	Hsa_circ_0011233
Location	chr1:31422979-31426828
Genomic Length	3849 bp
Spliced Seq Length	527 bp
Best Transcript	NM_014676
Gene Symbol	PUM1

## Data Availability

The data used and/or analyzed during the current study are available from the corresponding author on reasonable request.

## Supplementary Materials

The following supporting information can be downloaded at: https://www.mdpi.com/article/10.3390/genes15010124/s1, Figure S1: Amplification plot of the selected circRNAs (A). Report of Melting curve plot (B). Electrophoretic analysis of the circRNA products obtained by real-time PCR amplification (C). Figure S2: Representative illustration of PUM1 pre-mRNA and circular transcript (circPUM1) derived from back-splicing 15th, 16th, and 17th exons.
