# Supplementary material for: A Circular RNA Derived from the Pumilio 1 Gene Could Regulate PTEN in Human Cumulus Cells
Source: Genes (Basel). 2024 Jan 19;15(1):124. doi: 10.3390/genes15010124 (PMC10815046; doi:10.3390/genes15010124)

Supplementary data

Figure S1. Amplification plot of the selected circRNAs (A). Report of Melting curve plot (B). Electrophoretic analysis of the circRNA products obtained by Real Time PCR amplification (C).

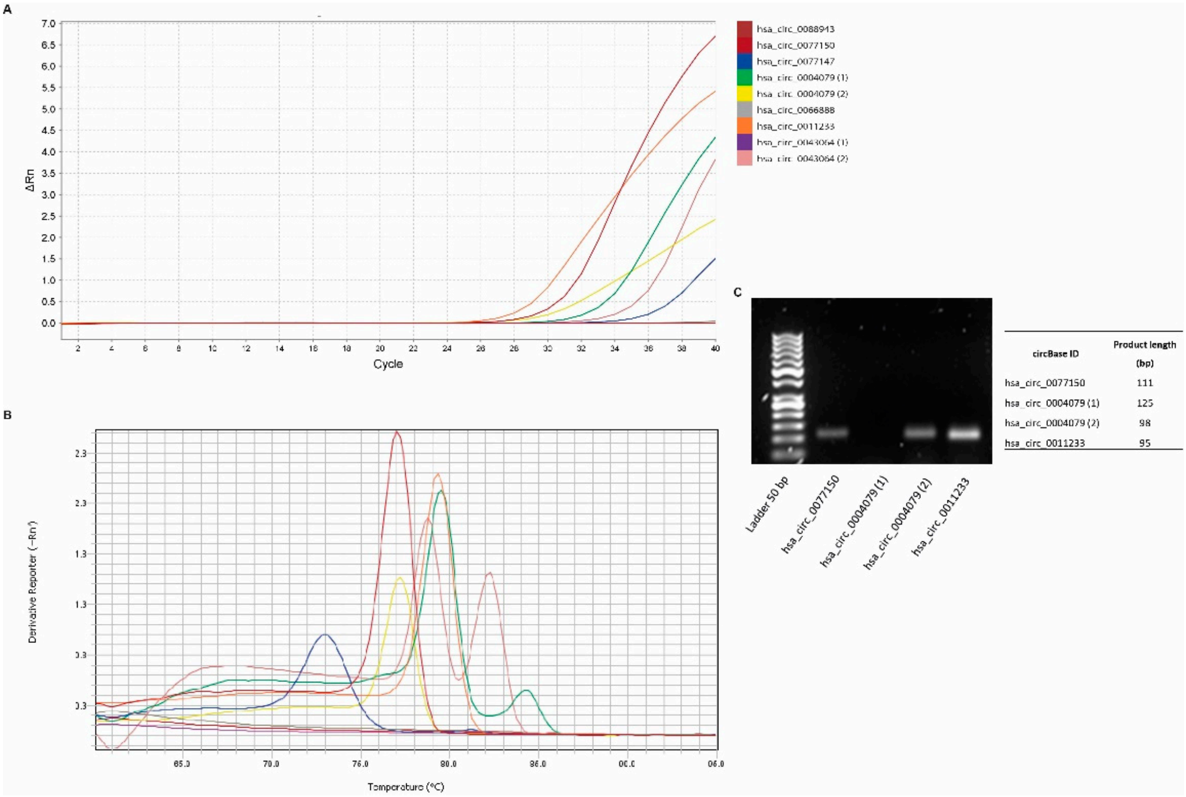

Figure S2. Representative illustration of PUM1 pre-mRNA and circular transcript (circPUM1) derived from back-splicing 15th, 16th and 17th exons.

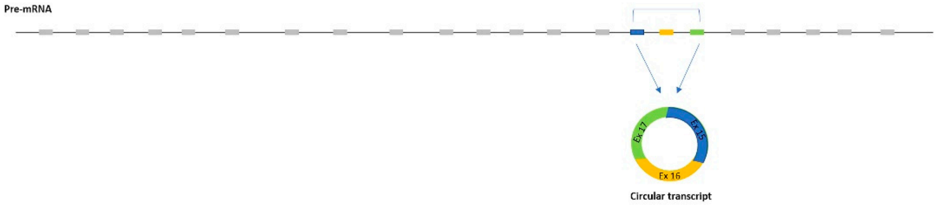

Supplement: Supplementary file 1 [file genes-15-00124-s001.zip › genes-2775237-supplementary.pdf]
